# Successful pregnancy in a patient with IgA nephropathy treated with telitacicept: a case report

**DOI:** 10.1186/s12884-024-06632-7

**Published:** 2024-06-17

**Authors:** Xinru Du, Geng Tian, Xuehong Lu

**Affiliations:** 1 Department of Nephrology, The Second Hospital of Jilin University, Changchun, China; 2 Department of Gynecology and Obstetrics, The Second Hospital of Jilin University, Changchun, China

**Keywords:** Case report, IgA nephropathy, Pregnancy, Telitacicept, Long-lived plasma cells

## Abstract

**Background:**

IgA nephropathy (IgAN) is the most common cause of primary glomerulonephritis, with complex pathogenic mechanisms involving abnormal B-cell activation. As a novel biologic agent, telitacicept inhibits both B-lymphocyte stimulating factor and a proliferation-inducing ligand. It also inhibits both B cells and plasma cells and the production of galactose-deficient IgA1 (Gd-IgA1) and its autoantibodies, thus exerting an immunosuppressive effect. Women with IgAN are at a higher risk of adverse pregnancy outcomes such as preeclampsia and miscarriage, especially those with uncontrolled massive proteinuria and advanced chronic kidney disease. Therefore, IgAN disease control before and during pregnancy is essential. Here, we report the case of a woman with IgAN who had a successful pregnancy with significant improvement and long-term remission after treatment with telitacicept. This is the first report of a pregnancy following exposure to telitacicept.

Case report.

**Conclusion:**

This report describes the efficacy of telitacicept in patients with IgAN and explores its value in women of childbearing age, suggesting effective and safe treatment options for women who wish to conceive.

## Background

IgA nephropathy (IgAN) is the most common cause of primary glomerulonephritis worldwide. Its occurrence is closely related to autoimmune mechanisms, as abnormally activated B lymphocytes play a key role in disease onset and progression [1]. B-lymphocyte stimulator (BLys/BAFF) and a proliferation-inducing ligand (APRIL) are important cytokines for B-cell maturation and differentiation [1]. Elevated levels of circulating Gd-IgA1 and autoantibodies against it are associated with an increased risk of disease progression [2]. Currently, specific treatments for IgAN are lacking; thus, targeting B-cell activation to reduce the production of Gd-IgA1 and its antibodies may provide a new therapeutic approach.

The incidence of IgAN in women is highest among those of reproductive age. Pregnant women with IgAN have a greater risk of adverse pregnancy outcomes, including miscarriage, a low birth weight infant, and preeclampsia than those without IgAN. Notably, proteinuria levels and chronic kidney disease (CKD) stage during pregnancy are closely related to adverse pregnancy outcomes [3]. Therefore, stabilizing proteinuria and kidney function during pregnancy may reduce the risk of complications in women with IgAN.

Telitacicept is a novel recombinant fusion protein consisting of the extracellular soluble portion of transmembrane activator and calcium modulator and cyclophilin ligand interactor receptor fused to the Fc portion of human IgG. It binds to and inhibits BLyS/BAFF and APRIL [4], thus targeting two components of the B-cell-mediated autoimmune response. This leads to the inhibition of Gd-IgA1 production and the suppression of disease activity and progression.

## Case presentation

**Fig. 1:**
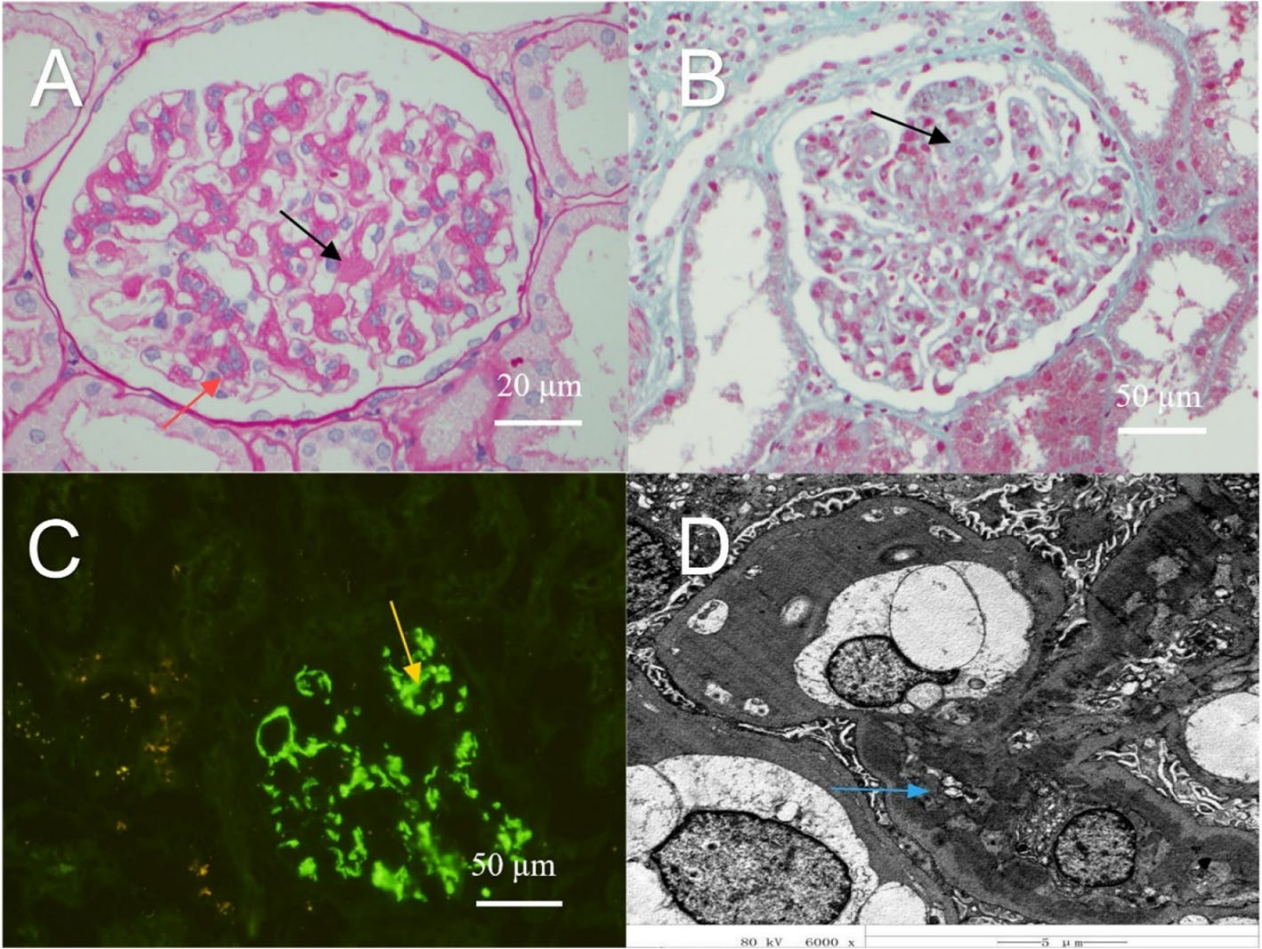
Pathological analysis of the renal biopsy tissue. **A** Periodic acid-Schiff stain; **B** Masson’s trichrome stain; **C** IgA immunofluorescence microscopy; **D** Electron microscopy. Black arrow: increased thylakoid stroma; red arrow: thylakoid cell hyperplasia; yellow arrow: deposition of IgA immune complexes by immunofluorescence; blue arrow: deposition of electron dense material in the thylakoid region

A 32-year-old female was found to have 3 + urinary protein levels in 2016 but did not receive any specific treatment. A follow-up examination in 2019 revealed a 3 + urinary protein level, 3 + urinary blood level, and serum creatinine level of 90 μmol/L. The outpatient physician prescribed the maximum tolerated dose of the renin–angiotensin–aldosterone system (RAAS) inhibitor valsartan (150 mg orally once daily). The patient was not seen again until September 2021, when she was hospitalized due to proteinuria and hematuria. Laboratory analysis revealed a serum creatinine level of 93 μmol/L, estimated glomerular filtration rate (eGFR) of 71.3 mL/min, 3 + urinary blood level, and 3 + urinary protein level. The patient’s blood pressure was normal, and renal ultrasonography revealed no abnormalities. The patient had a history of pulmonary tuberculosis at 15 years of age; however, chest computed tomography during hospitalization showed no disease activity. Based on these results, a renal biopsy was performed, revealing the following immunofluorescence findings: IgA (+ +), IgM (-), IgG (+ -), C3 (+ +), and C4 (-). Light microscopy revealed 23 intact glomeruli, 3 globally sclerosed glomeruli, 1 cellular crescent, and 1 small cellular crescent, all with segmental sclerosis, and 2 adhesions. Diffuse mild-to-moderate mesangial cell proliferation, severe focal segmental proliferation, increased mesangial matrix, and proliferation of endothelial cells in the segmental loop were observed. Focal tubular epithelial cell granulation, vacuolar degeneration, and atrophy, as well as protein casts, were visible. Interstitial fibrosis and inflammatory cell infiltration were observed. A few small arteries showed slight thickening of the vessel wall. Eosinophilic deposits were also observed in the mesangial area. The pathological diagnosis was IgAN (Lee classification, grade 3; Oxford classification, M1E1S1T0) (Fig. 1). The patient was prescribed oral prednisone acetate (50 mg/day).

In October 2021, the patient visited the outpatient clinic again, with an increased serum creatinine level (126 μmol/L), a 24-h urinary protein level of 4.67 g, a urinary erythrocyte level of 1274/μL, urinary leukocytes ( +), increased white blood cell counts in peripheral blood, and an elevated neutrophil percentage, indicating urinary tract infection. Changes in blood and urine test markers associated with the initial hospitalization and re-hospitalization of the patient for renal biopsy are shown in Table 1.

**Table 1:** Routine blood, renal function, and urinalysis results from two hospitalizations

**Urinalysis parameter**	**First hospitalization (biopsy)**	**Re-hospitalization**	**Reference range**
White blood cell count	6.7×10^9^	10.9×10^9^	3.5–9.5×10^9^
GR% granulocyte	37.9	88.8	40.0–75.0
Creatinine	93 µmol/L	124 µmol/L	41–73
Blood urea nitrogen	5.18 mmol/L	6.64 mmol/L	2.6–7.5
Estimated glomerular filtration rate	71.3 mL/min	50 mL/min	>90
IgG	14 g/L	12.38 g/L	7.51–15.6
IgA	4.42 g/L	3.71 g/L	0.82–4.53
IgM	1.34 g/L	1.32 g/L	0.46–3.04
C3	62.3 mg/dL	61.1 mg/dL	79–152
C4	15.9 mg/dL	15.2 mg/dL	16–38
C reactive protein	0.01 mg/L	0.69 mg/L	<5.0
24-h urinary protein	1.71 g	4.67 g	0.0–0.15
Occult	3+	3+	
LEU	-	1+	
Protein	2+	3+	
Urine red blood cell count	135/µL	1274/µL	0.0–17.6
Urine White blood cell count	8/µL	145µL	0.0–25

The patient was admitted for the second time with significant elevation of creatinine, urinary protein, urinary erythrocytes, and leukocytes in the blood, and urinary tract infection could not be excluded. The infection might have been related to the long-term administration of steroid therapy. Antibiotics administration successfully controlled the infection, and we reexamined her urine and renal function 1 week after admission. Urinary protein levels and erythrocyte counts had decreased from 4.67 g to 2.02 g and from 1274/μL to 505/μL, respectively, whereas her serum creatinine levels remained relatively stable. We could not ascertain whether the patient's significantly decreased urinary protein level and erythrocyte count were caused by the control of urinary tract infection. In addition, the patient expressed a desire to avoid the side effects of high-dose steroids use, as well as a strong desire to conceive and, therefore, hoped that the disease could be controlled as quickly as possible.

**Fig. 2:**
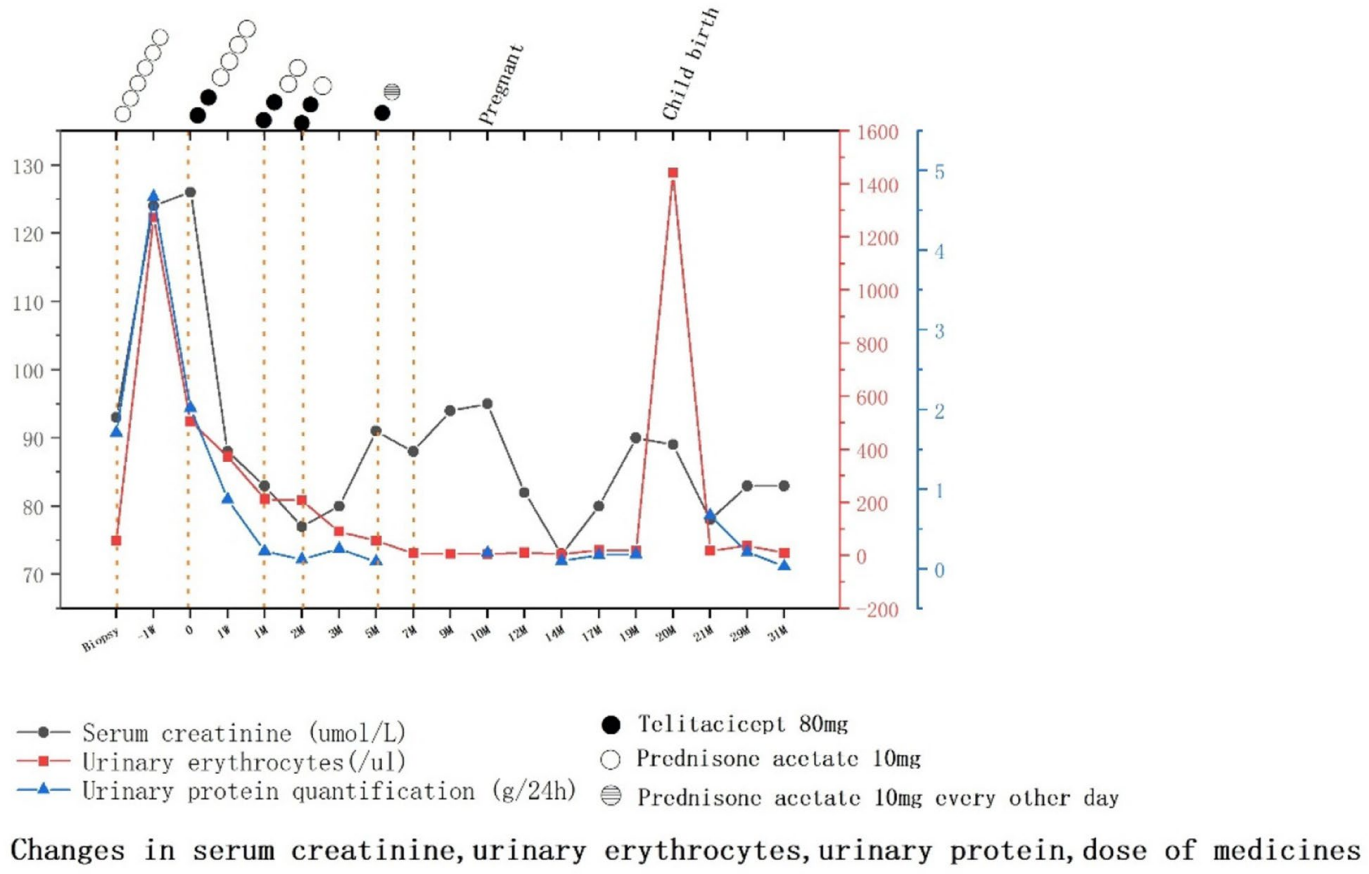
Changes in serum creatinine, urinary erythrocytes, and urinary protein levels, and medications administered

After 1 week of admission for infection control, we treated her with steroid therapy combined with telitacicept and advised her to undergo regular outpatient follow-up. After 1 week of telitacicept treatment, the patient's proteinuria, hematuria, and creatinine level decreased significantly, with proteinuria decreasing from 2.02 g to 0.87 g, urinary erythrocytes decreasing from 505/μL to 373/μL, and blood creatinine decreasing from 124 μmol/L to 88 μmol/L. Since then, her 24-h proteinuria had remained stable at less than 0.5 g. We adjusted the dosage of steroids and telitacicept according to the patient's condition. Following the successful reduction of the dose of prednisone acetate to 10 mg, the dosage was slowly tapered to 0 mg. The treatment course is presented in Fig. 2. In early June 2022, the patient stated that she planned to become pregnant because of her stable condition. She received her last dose of telitacicept on June 17, 2022; at the same time, the prednisone acetate was also discontinued. In accordance with the manufacturer’s guidelines, we recommended the patient to wait for 4 months from the date of telitacicept discontinuation before attempting to conceive. However, the patient became pregnant 3 months after stopping the medication. After a comprehensive evaluation by the obstetrician and nephrologist and after explaining the potential risks to the patient, the patient requested to continue the pregnancy and was told to visit the nephrologist and the obstetrician for follow ups. The patient's 24 h proteinuria remained consistently below 0.5 g during pregnancy, her blood creatinine was stable at 70–95 μmol/L, her urine erythrocytes were stable at 5–18/μL, and her blood pressure was within the normal range, weight gain during pregnancy 15 kg.

On June 5, 2023 (39 week and 3 days of pregnancy), she delivered a boy, weighing 2750 g and measuring 41 cm, with an Apgar score of 9 at 1 min and 10 at 5 min. At 1 month postpartum, the patient's urinary protein level was 0.67 g/24 h, creatinine level was 78 μmol/L, and erythrocyte count was 18/μL. The proteinuria level was elevated compared with the previous one, which might be related to postpartum secretions such as lochia. Considering the patient's need for breastfeeding, we administered hydroxychloroquine 200mg/dose twice daily oral maintenance therapy. Thereafter, the patient's condition stabilized. The last follow-up in our hospital was on May 21, 2024, and the patient's proteinuria was 0.03 g/24 h and her blood creatinine level was 83 μmol/. Urine erythrocyte count was 9.41/μl. At this time the patient had stopped breastfeeding and we reduced the hydroxychloroquine dose to 200 mg daily and added irbesartan 150 mg daily.From the time the patient started telitacicept treatment until a long time after delivery, she had no recurrence of the disease, and her condition was significantly controlled. The patient's specific follow-up indicators are shown in Table 2. Our research center is conducting a study on Elabela combined with Bayesian Stochastic Modelling to assess the prognosis of IgAN (The Second Hospital of Jilin University, No. 2022LC116). We evaluated patients with this model and found that the prognosis of the disease significantly improved with the administration of telitacicept. The study was approved by the Ethics Committee of the Second Hospital of Jilin University (Ethics No. 2021174) and followed the guidelines of the Declaration of Helsinki.

**Table 2:** Laboratory data of patients before and after treatment with telitacicept

	Creatinine μmol/L	BUN mmol/L	eGFR mL/min	IgG g/L	IgA g/L	IgM g/L	C3 mg/dl	C4 mg/dl	CRP mg/L	24 h-UP g/d	UWBC /μL	Protein g/L	URBC μL
Biopsy	93	5.18	71	14	4.42	1.34	62.3	15.9	0.01	1.71	8	2 +	56
▲-1 week	124	6.64	50	12.38	3.71	1.32	61.1	15.2	0.69	4.67	145	3 +	1274
*0	126	5.44	49	—	—	—	—	—	< 3.3	2.02	12	2 +	505
1 week	88	5.29	76	10.85	3.41	1.29	61.7	16.1	< 3.3	0.87	24	1 +	373
1 month	83	5.16	81	7.69	2.32	1.1	63.5	19.3	< 3.3	0.22	88	0	212
2 months	77	4.06	90	6.5	1.69	0.802	68.6	23	< 3.3	0.12	18	0	208
3 months	80	4.21	85.5	6.27	1.6	0.782	87.6	25.6	< 3.3	0.25	22	1 +	91
5 months	91	3.66	73.2	5.7	1.39	0.533	76.2	19.1	< 3.3	0.09	5	0	56
**7 months	88	5.64	75.7	6.86	1.59	0.521	73.8	17.4	< 3.3	—	0	0	8.82
9 months	94	5.45	70	6.86	1.51	0.487	60.8	15.3	< 3.3	—	0	0	6.47
Δ10 months	95	6.28	69	—	—	—	—	—	< 3.3	0.2	0	0	5.29
12 months	82	4.76	82.4	8.77	1.98	0.655	61.2	15.7	< 3.3	—	5.1	0	11
14 months	73	3.27	94.8	8.41	2.03	0.785	62.7	15.8	< 3.3	0.1	4.3	0	5.29
17 months	80	4.24	84	9.97	2.59	0.98	61.1	15.5	< 3.3	0.17	14.2	0	20.6
19 months	90	3.73	72.9	10.41	2.97	1.04	61.3	15.6	< 3.3	0.18	54.8	0	17.7
ΔΔ20 months	89	3.79	74.1	—	—	—	—	—	—	—	133.7	1 +	1441.8
21 months	78	4.31	86.9	10.69	3.05	1.06	62.2	15.8	< 3.3	0.67	10.1	1 +	18
29 months	83	6.91	80.6	11.69	3.5	1.18	62.3	16	< 3.3	0.21	3.9	1 +	37.06
31 months	83	5.36	80.1	11.50	3.03	1.29	68.0	17	< 3.3	0.03	5.88	0	9.41

*BUN* Blood urea nitrogen, *eGFR* Estimated glomerular filtration, *CRP* C reactive protein, *24 h-UP* 24-h urinary protein, *URBC* Urinary Red Blood Cell Count, *UWBC* Urinary White Blood Cell Count

▲Urinary infection * Telitacicept START ** Telitacicept STOP Δ Pregnant ΔΔ Childbirth

Taking the start of telitacicept treatment as a time reference. Unfortunately, our follow-up information was partially missing immunoglobulin and complement at the start of treatment and the 10th month after telitacicept. At month 20 after telitacicept treatment, we only had laboratory data related to her hospitalization in the obstetrics department, and therefore immunoglobulin and complement tests were missing

The patient did not undergo 24-h quantitative urinary protein testing in the 7th, 9th, and 12th month after treatment with telitacicept because routine urinalysis revealed trace amounts of urinary proteins. At the 20th month after treatment, which coincided with the time of delivery, the patient did not undergo urinary protein testing because of the interference caused by the delivery, and at this time, the significant elevation of urinary erythrocytes was considered to be caused by the delivery.

## Discussion and conclusions

In the present case, the patient did not achieve complete remission after treatment with valsartan and experienced urinary tract infection after oral corticosteroid treatment. However, when treatment with telitacicept was introduced, proteinuria, hematuria, and serum creatinine levels significantly decreased within a short period, and successful conception was achieved after drug discontinuation. Disease relief was also achieved before, during, and long after pregnancy. This provides a new treatment approach for women who wish to become pregnant, as it may cause rapid improvement of the disease and, therefore, reduce the risk of adverse pregnancy outcomes.

Treatment of IgAN is challenging in women of childbearing age. However, a higher incidence of adverse pregnancy outcomes has been observed in patients with IgAN, even those with preserved kidney function. An analysis of 820 pregnancies in 557 women revealed that 88.3% resulted in live births, 14.2% in preterm births, 13.1% in low birth weight infants, 8.6% in preeclampsia, and 49.1% in cesarean sections [3, 5–8]. Meanwhile, several studies have shown that elevated proteinuria and creatinine levels before and during pregnancy may be significant risk factors for adverse pregnancy outcomes in patients with IgAN [3, 7, 8]. Therefore, stabilizing kidney function and reducing urinary protein levels before and during pregnancy are critical. As our patient is a young woman who presented desire to conceive needs and had an elevated blood creatinine level, we discontinued immunosuppressive drugs with teratogenic or potentially teratogenic risks as well as damaging effects to the renal function. In addition, these drugs have inherent limitations, including nonspecific targeting, numerous adverse effects, high relapse rates, and long treatment cycles. Therefore, there is a need for a drug that can achieve significant disease remission before pregnancy and allows patients to conceive during remission.

Targeting the B-cell pathway to inhibit Gd-IgA1 and antibody production is a therapeutic strategy based on the "quadruple whammy hypothesis" of IgAN pathogenicity. Elevated BAFF/Blys and APRIL are thought to correlate with serum Gd-IgA1 levels, and a positive correlation between serum BLys/BAFF levels and glomerular tunica IgA deposition density and serum IgA1 levels in IgAN patients has been reported [9, 10]. Kim et al. reported that antibody-targeting of APRIL resulted in decreased proteinuria, serum IgA levels, and glomerular IgA deposition in a mouse model of IgAN [11]. Additionally, Muto et al. reported that APRIL expression was significantly increased in the tonsils of patients with IgAN; patients with APRIL overexpression responded well to tonsillectomy, which reduced serum Gd-IgA1 levels, confirming the involvement of APRIL in IgAN progression [12]. However, conventional B-cell depletion therapies such as rituximab do not seem to be effective in reducing Gd-IgA1 and its antibody levels [13]. This is because it did not affect the survival of CD20-negative long-lived plasma cells in vivo. An increasing body of research indicates that long-lived plasma cells play a crucial role in the chronicity, refractoriness, and recurrence of autoimmune diseases [14, 15]. Without the elimination of the auto-reactive memory generated by the plasma cells, the disease is unlikely to be cured, and therefore, permanent medication is often required. Nevertheless, the potential long-term negative effects of such permanent medication cannot be ignored [13, 16, 17]). BAFF or APRIL is thought to be critical in maintaining plasma cell survival for long periods of time [17]. However, several studies have shown the redundancy of BLyS/BAFF and APRIL; blocking both cytokines is therefore necessary to impair long-lived plasma cell survival [18, 19].

Telitacicept inhibits the development and survival of long-lived plasma cells and mature B cells through dual-targeted inhibition of BLys/BAFF and APRIL, reduces IgA1 secretion, reduces Gd-IgA1 production, inhibits the formation of anti-Gd-IgA1 autoantibodies, and reduces immune-complex deposition in the glomerular plasma membrane. Additionally, compared with other immunosuppressants, telitacicept can achieve long-term remission, perhaps because it can affect the survival of long-lived plasma cells, but we could not test these hypotheses because we did not monitor peripheral blood B-cell subsets and plasma cell counts throughout the course of the disease.

There are limited safety studies of telitacicept in pregnancy. Before the drug was marketed, the manufacturer conducted reproductive toxicity studies in rats and rabbits, which showed no maternal toxicity or embryo-fetal developmental toxicity and no teratogenic effects in either preconception or gestational drug exposure. In addition, a phase IIb clinical study of telitacicept in patients with SLE revealed an unintended pregnancy in a woman after 34 drug exposures at a gestational week of 7w5d + , after which she successfully gave birth to a healthy male child [20]. In our study, the patient did not follow the manufacturer's prescribed discontinuation time for pregnancy and subsequently delivered without incident. In summary, it may be time to re-emphasize the safety of using telitacicept before, during, and even throughout pregnancy. More studies are needed in the future to explore its benefits and risks during pregnancy.

This is the first report of pregnancy following telitacicept exposure. The patient experienced sustained control and remission of the disease before, during, and for more than 2 years after pregnancy. The long-term remission and control of the disease with telitacicept helped minimize pregnancy-related adverse outcomes, and the value of this novel biologic for women of childbearing age needs to be considered in the future.

## Data Availability

No datasets were generated or analysed during the current study.

## Abbreviations

IgAN: IgA nephropathy
BLys/BAFF: B-lymphocyte stimulator
APRIL: Proliferation-inducing ligand
CKD: Chronic kidney disease
RAAS: Renin–angiotensin–aldosterone system
eGFR: Estimated glomerular filtration rate
